# Drug-Induced Hypersensitivity Syndrome: A Clinical, Radiologic, and Histologic Mimic of Lymphoma

**DOI:** 10.1155/2018/7037352

**Published:** 2018-10-21

**Authors:** Faaria Gowani, Bradley Gehrs, Teresa Scordino

**Affiliations:** Department of Pathology, The University of Oklahoma Health Sciences Center, 940 Stanton L. Young Boulevard, BMSB 451, Oklahoma City, OK 73104, USA

## Abstract

Drug-induced hypersensitivity syndrome (DIHS; also known as drug reaction with eosinophilia and systemic symptoms, or DRESS) is a rare, potentially life-threatening condition that typically presents 2–8 weeks after drug exposure with fever, rash, organ dysfunction, and lymphadenopathy. Here, we describe the case of an 18-year-old African American female who presented with cervical lymphadenopathy, fevers, and a macular rash. A PET scan showed diffuse hypermetabolic lymphadenopathy suggestive of lymphoma, with involvement of the spleen and kidneys. The clinical history, imaging, and biopsy findings initially raised concern for a malignant process, with a differential diagnosis including classic Hodgkin's lymphoma and T-cell lymphoma. However, the morphologic and immunophenotypic features were not entirely typical for those diagnoses. The patient was ultimately diagnosed with DIHS after it was learned that she recently had been treated with minocycline, a medication previously implicated in causing DIHS.

## 1. Case Presentation

An 18-year-old African American female with a history of hyperthyroidism presented to our facility with fever, chills, body aches, significant cervical lymphadenopathy, facial edema, and a progressive (eventually generalized) macular morbilliform rash. She reported that elevated transaminases had been noted during a recent visit with her endocrinologist. Her CBC included a WBC count of 8,600 mm^3^, hemoglobin of 12.4 g/dl (MCV 77.8 fL), and platelet count of 261,000 mm^3^. Ferritin was markedly elevated (1229.6 ng/mL); serum iron, TIBC, and iron saturation were decreased, suggestive of anemia of chronic disease. AST was elevated to 127 units/mL, and ALT was elevated to 239 units/mL. Hemoglobin electrophoresis revealed normal adult hemoglobin. A rapid HIV test was nonreactive. PCR testing of peripheral blood was negative for EBV and HHV-6. Multiple blood cultures were negative.

**Figure 1:**
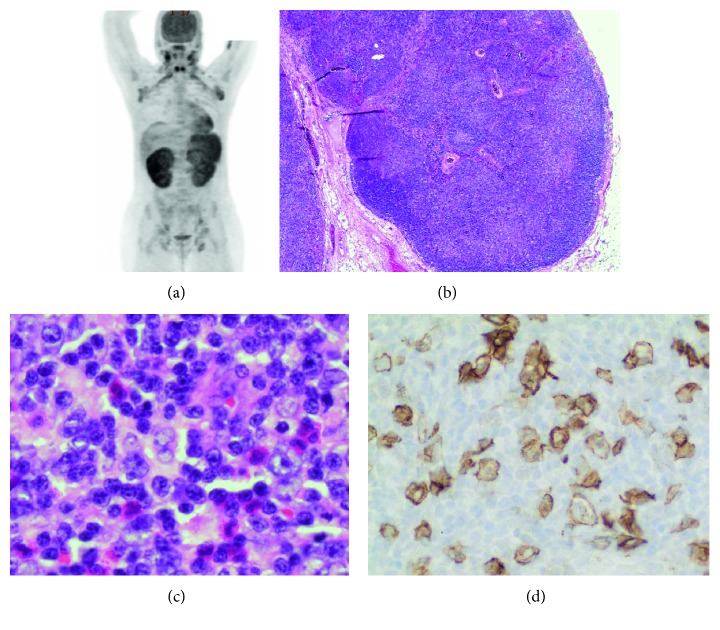
A PET/CT scan showed hypermetabolic lymphadenopathy involving cervical, supraclavicular, axillary, pelvic, and inguinal nodes and findings consistent with malignant infiltration of the bilateral kidneys and spleen (a). Low-power examination of the lymph node showed distortion of the lymph node architecture, with expansion of the paracortex (b) (H&E, 40X magnification). High-power examination of the paracortex showed a mixed inflammatory infiltrate with increased eosinophils and scattered large cells (c) (600X). An immunohistochemical stain for CD30 highlighted a patchy increase in large immunoblasts (d) (400X).

Abdominal ultrasound showed splenomegaly and enlarged porta hepatis lymph nodes. A PET scan revealed diffuse hypermetabolic lymphadenopathy involving cervical, supraclavicular, axillary, pelvic, and inguinal nodes, as well as findings consistent with malignant infiltration of the bilateral kidneys and spleen (Figure 1(a)). Although the possibility of a drug reaction had been in the differential diagnosis prior to the imaging studies, the degree and extent of the imaging abnormalities raised clinical concern for a malignant process.

Due to the concern for malignancy, axillary lymph node and bone marrow biopsies were performed. Examination of the bone marrow showed that it was appropriately cellular for age (∼80%), with maturing trilineage hematopoiesis, polyclonal plasmacytosis, and eosinophilia. Scattered small T-cell aggregates were present. Flow cytometric immunophenotyping did not detect any abnormal lymphoid populations. No evidence of malignancy was identified.

Histologic examination of the lymph node revealed mostly preserved, but significantly distorted, nodal architecture with expansion of the paracortex by a mixed infiltrate of small lymphocytes, eosinophils, histiocytes, plasma cells, and scattered large atypical lymphoid cells, including occasional Reed–Sternberg-like cells. Secondary follicles were largely absent (Figures 1(b) and 1(c)). A few apoptotic bodies and pigment-containing histiocytes were identified.

Immunohistochemical stains for CD20, PAX5, CD79a, OCT2, and BOB-1 highlighted the B-cell population in the cortex that was largely confined to primary follicles. CD23 highlighted irregularly expanded follicular dendritic cell meshworks. CD3 highlighted numerous T cells in the paracortex and interfollicular areas. CD15 highlighted granulocytes. CD30 highlighted scattered large immunoblasts, including rare Reed–Sternberg-like cells (Figure 1(d)); no sheets of positive cells were seen. MUM1 was positive in plasma cells, predominantly in the medullary cords and sinuses. ALK immunostaining and EBV in situ hybridization (EBER) were negative.

The lymph node findings, including architectural distortion, expanded follicular dendritic cell meshworks, eosinophilic infiltrate, and proliferation of large CD30-positive lymphoid cells raised concern for a malignant process such as T-cell lymphoma or classic Hodgkin's lymphoma; however, the morphologic and immunophenotypic features were not entirely typical for those diagnoses. Molecular analysis of the lymph node did not detect any monoclonal IGH or IGK gene rearrangement or T-cell receptor gene rearrangement.

Two days after the lymph node biopsy was performed, the patient's WBC count had risen to 24,100/mm^3^. Examination of the peripheral blood smear revealed neutrophilia, atypical lymphocytes, and mild relative eosinophilia, with an increased absolute eosinophil count of 1,400/mm^3^. On further review of the patient's history, it was noted that the patient had received a course of minocycline to treat folliculitis, beginning approximately five weeks prior to admission and ending four days prior to admission.

After consideration of all of the available information, a diagnosis of DIHS was made. The minocycline was discontinued permanently. Following treatment with prednisone, the patient's rash, leukocytosis, and lymphadenopathy gradually resolved.

## 2. Discussion

Drug-induced lymphadenopathy was described as early as the 1920s [1]. In 1959, Saltzstein and Ackerman reported a case series and literature review of drug-induced lymphoma-like adenopathy, a syndrome that included fever, rash, lymphadenopathy, and variable hepatosplenomegaly, in patients treated with anticonvulsant drugs [1]. Since then, the condition has been variably known as drug-induced pseudolymphoma, drug reaction with eosinophilia and systemic symptoms (DRESS) syndrome, drug-induced delayed multiorgan hypersensitivity syndrome (DIDMOHS), and drug-induced hypersensitivity syndrome (DIHS) [2].

A number of drugs have been implicated in development of DIHS, including phenytoin, carbamazepine, other anticonvulsants, minocycline, sulfasalazine, allopurinol, and dapsone; aromatic anticonvulsants are among the most common inciting agents [2–4]. The etiology of DIHS is incompletely understood. Proposed contributing factors include a possible hapten-like reaction between the drug and a host protein [5] and deficiencies in drug-metabolizing enzymes, leading to accumulation of metabolites that may cause cell damage and induce an immune response [2, 4, 6]. Activation of T cells and macrophages leads to release of cytokines, including IL-6 and tumor necrosis factor [2, 3]. An inherited predisposition has been proposed, based on the observation that the risk of developing DIHS appears to be increased in individuals with certain HLA haplotypes [2, 6]. An association with reactivation of HHV-6 and other herpesviruses has been observed. Viral reactivation may occur several weeks after the onset of symptoms [2, 4, 6, 7].

Development of symptoms typically occurs 2–8 weeks after exposure to the medication, though earlier onset may be seen in patients with previous exposure to the offending agent [3]. Fever and skin rash are the most common presenting symptoms; the rash is typically morbilliform, but skin manifestations are variable and may be absent in a subset of patients [6]. Evidence of extracutaneous organ dysfunction is often present and may manifest as transaminitis, renal insufficiency, pneumonitis, myocarditis, or neurologic abnormities [3, 6]. The majority of DIHS patients have either localized or generalized lymphadenopathy [6]. Peripheral blood abnormalities, including leukocytosis with reactive lymphocytosis and/or eosinophilia, are common [3, 6]. Hypogammaglobulinemia is common, though hypergammaglobulinemia has also been reported [4, 8, 9]. Other laboratory abnormalities may include elevations in LDH and ferritin [3]. Ferritin levels may correlate with disease severity [10]. Elevated ferritin levels may also be seen in association with hemophagocytic syndrome, which is a rare complication of DIHS [11, 12]. The clinical and laboratory findings may raise a differential diagnosis that includes severe infection, malignancy, or autoimmune disease; in particular, adult-onset Still's disease shows several overlapping diagnostic features, including fever, lymphadenopathy, leukocytosis, and elevated ferritin levels [13].

A scoring system has been proposed to classify cases of definite, probable, or possible DIHS in hospitalized patients, based on clinical manifestations (fever, lymphadenopathy, skin rash, duration of symptoms, and organ involvement), laboratory abnormalities (eosinophilia, atypical lymphocytes, and laboratory evidence of organ dysfunction), and exclusion of rheumatologic disease and other alternate etiologies [14]. HHV-6 reactivation has also been suggested as a diagnostic criterion [7].

Lymph node biopsy findings in DIHS may vary based on the timing of disease onset and the timing of biopsy procurement. In the acute phase, there is enlargement of the affected lymph nodes, with variable follicular hyperplasia and expansion of the paracortex, with an infiltrate of T cells, eosinophils, and immunoblasts. Vascular proliferation and vasculitis may be present. In later onset disease, follicles may be atrophic and eosinophils may be less prominent [3]. Given that a skin rash is usually present, dermatopathic changes may also be seen [3].

The lymph node biopsy findings in DIHS may raise concern for lymphoma. Marked expansion of the paracortex may distort the lymph node architecture to the point where it is unrecognizable on hematoxylin and eosin-stained slides. The presence of a mixed inflammatory background with eosinophils may suggest a differential diagnosis of classic Hodgkin's lymphoma. Vascular proliferation and eosinophilia may also raise concern for a T-cell lymphoma, such as angioimmunoblastic T-cell lymphoma (formerly known as angioimmunoblastic lymphadenopathy) [15]. Decreased expression of pan-T-cell antigens, including CD3 and CD7, has been reported in some cases of DIHS [16], which may further suggest a diagnosis of T-cell lymphoma (Table 1). Skin biopsies, if performed, may also show findings concerning for a malignant process. Stephan et al. reported a case of lamotrigine-induced DIHS wherein a skin biopsy showed a proliferation of CD30-positive cells, concerning for a CD30-positive cutaneous lymphoma [17].

Treatment of DIHS involves removal of the offending drug. Corticosteroid therapy is commonly used, with addition of IVIG and/or plasmapheresis in some cases [4]. Gradual tapering of corticosteroid therapy has been recommended to prevent recurrence of symptoms [7].

## 3. Conclusion

DIHS should be included in the differential diagnosis of patients presenting with fever, rash, and lymphadenopathy. Imaging findings may be striking and raise concern for malignancy. The clinical and laboratory features of DIHS may evolve over time, and the classic features may not all be present at initial presentation. This case emphasizes the importance of incorporation of the patient's clinical and medication history in the interpretation of lymph node biopsy specimens. Awareness of the clinical, radiologic, and pathologic findings of DIHS is essential to avoid a misdiagnosis of malignancy.

## Figures and Tables

**Table 1:** Comparison of clinical and pathologic features of DIHS, classic Hodgkin's lymphoma, and angioimmunoblastic T-cell lymphoma. IGH: immunoglobulin heavy chain. TCR: T-cell receptor.

	Drug-induced hypersensitivity syndrome (DIHS)	Classic Hodgkin's Lymphoma (CHL)	Angioimmunoblastic T-cell lymphoma (AITL)
*Clinical presentation*			
** **Age	Any age	Young adult or bimodal distribution, depending on subtype	Middle age to elderly
** **Lymphadenopathy	Present	Present	Present
** **B symptoms	Present	Present	Present
** **Skin rash	Present	Absent	Frequently present
** **History of drug exposure	Present	Absent	Absent
** **Prognosis	Excellent	Good	Poor

*Morphology*			
** **Reed–Sternberg-like cells	May be present	Present	May be present
** **Lymph node architecture	May be significantly distorted, but generally at least partially preserved	Effaced	Variably effaced; residual germinal centers may be present
** **Inflammatory cells (i.e., eosinophils)	Present	Present	Present
** **Vascular proliferation	Present	Absent	Present

*Immunophenotype*			
** **CD30 (in large cells)	+	+	+
** **CD15 (in large cells)	−	+	−
** **CD45 (in large cells)	+	−	+
** **T-cell population	Usually normal; may show diminished expression of one or more pan-T-cell markers	Normal; CD4:CD8 ratio usually increased	Abnormal; usually CD4 positive, with expression of follicular helper T-cell markers (PD1, CXCL13, BCL6, and CD10)

*Molecular abnormalities*	No clonal IGH or TCR gene rearrangements	Clonal IGH gene rearrangement may be detected, particularly in microdissected tissue	Clonal rearrangement of TCR gene
